# The Attitude of Great Writers Towards Disease

**Published:** 1892-11-12

**Authors:** 


### The Attitude of Great Writers Towards Disease.

### XV.—THE SICK IN UTOPIA.

It has been a favourite amusement of many philosophers to act the part of Providence towards an ideal state of their own framing. They would all prefer, of course, to people their states with perfectly healthy men and women, and to keep them so. But they dare not ignore the stern facts of life, and accordingly we find among all these dreams of perfection some account taken of inevitable sickness and decay. The change in tone towards the sick is well worth noting, which may thus be traced between a period nearly 3,000 years ago and an age not greatly preceding our own.

Lycurgus, as he was the first who designed a commonwealth, was the only one of the number whose theories have stood the test of experience. Very minute were his regulations for ensuring the physical strength of his people, starting with the careful selection and destruction of all weakly children. For the general maintenance of health in Sparta he seems to have largely relied on the efficacy of three agents which have since somewhat failed perhaps to sustain their reputation as sanitary influences, viz., dirt, hunger, and cold. No doubt, however, the result tended towards "the survival of the fittest;" and in a state where bodily infirmity was the lowest form of disgrace, there can have been no temptation to preserve lives maimed by disease or age.

In Plato's Republic the tone adopted towards the sick is hardly less censorious than that of Lycurgus. Plato is strongly of opinion that no one has any right to be ill. "And do you not hold it disgraceful to require medical aid, unless it be for a wound or an attack of illness incidental to the time of year—to require it, I mean, owing to our laziness, and the life we lead, and to get ourselves so stuffed with humours and wind, like quagmires, as to compel the clever sons of Asclepius to call diseases by such names as flatulence and catarrh?"

And if colds or indigestion are reckoned as a fault, continued illness is a crime. "In all well regulated communities no one has leisure to spend his life as an invalid in the doctor's hands." The art of the physician in Plato's opinion, can only be exercised lawfully on those of the citizens whose bodily constitutions are sound and good. "As for the constitutionally diseased and the intemperate, they thought the existence of such a man no gain either to himself or to others, believing that their art was not meant for persons of that sort, and that it would be wrong to attempt their cure, even if they were richer than Midas."

What a leap from Plato to Sir Thomas More. We have never yet attained the high water mark of his Utopia in regard to the duty of the State towards the poor, the aged, and the sick. "They take more care of their sick than of any others; these are lodged and provided for in public hospitals. They have belonging to every town four hospital that are built without their walls, and are so large that they may pass for little towns. By this means, if they had ever such a number of sick persons, they could lodge them conveniently, and at such a distance, that such of them as are sick of infectious diseases may be kept so far from the rest that there can be no danger of contagion. The hospitals are furnished and stored with all things that are convenient for the ease and recovery of the sick, and those that are put in them are looked after with such tender and watchful care, and are so constantly attended by their skilful physicians, that as none is sent to them against their will, so there is scarce one in a whole town that, if he should fall ill, would not choose rather to go thither than be sick at home."

Yet in the treatment of incurable disease Sir Thomas More is nearly of accord with the Greek philosophers. "For those who are taken with fixed and incurable diseases they use all possible ways to cherish them and to make their lives as comfortable as possible; but when any is taken with a torturing and lingering pain, so that there is no hope either of recovery or ease, the priests and magistrates come and exhort them that they should choose rather to die since they cannot live but in much misery. Such as are wrought on by these persuasions either starve themselves of their own accord, or take opium, and by that means die without pain. But no man is forced on this way of ending his life."

It will be noticed that More abstains from particulars in his care of the sick. It is to be well done—as well as possible—but he does not say how. Now Bacon in his "New Atlantis" goes a step farther. He mentions the infirmary in his state with "cells, very neat ones, having partitions of cedar wood," but he devotes a great deal more space to the discussion of new methods, not only of healing, but of enlarging the bounds of medical science. Not the least curious of his devices are the vast caves which are artificially formed under certain mountains, "for curing of some diseases and the prolongation of life." Artificial wells and fountains "tincted upon vitriol, sulphur, steel, brass, lead, nitre, and other minerals" were to be tried; also "fair and large baths of several mixtures for the cure of diseases," with "certain chambers of health where we qualify the air as we think good and proper for the cure of divers diseases and the preservation of health." He appears to have been to some extent an advocate for vivisection. "We have also enclosures of beasts and birds, which we use for dissections and trials, that thereby may take light what may be wrought upon the body of man. Wherein we find many strange effects; as continuing life in them, though divers parts which you account vital be perished and taken forth; resuscitating of some that seem dead in appearance, and the like. We try also all poisons and other medicines upon them, as well of chirurgery as physic." The like regard to experiment is shown in the "dispensatories or shops of medicines." In fact, Bacon evidently revelled in the contemplation of a nation dedicated to research on this as on every other conceivable subject.

Campanella, contemporary with Bacon, reveals in a striking manner the essential points of difference between English and Italian modes of thought and practice. The City of the Sun is a curiously simple region, and comparing its arrangements for the sick with the ideals of More and Bacon, we are driven to consider whether the deficiencies in modern Italian hospital work, lately revealed in Mr. Burdett's "Hospitals of the World," may not be due as much to the vagueness of Italian thinkers as to any want of zeal on the part of a government nobly emulous of what is best in its reforms. Here in the City of the Sun we have no organisation for the care of the sick, no attempt to advance on the lines of scientific experiment; we are in the old atmosphere again of guess-work and tradition. Diet is to cure most things (this is thoroughly Italian), and cleanliness (evidently a new idea) is to obviate what cannot be cured. Still, there are many truly sensible suggestions, especially in view of the very different and often entirely brutal methods then in vogue. "They treat fevers with pleasant baths and with milk foods, and with a pleasant habitation in the country, and by gradual exercise." "They cure hot fevers with cold potations of water, but slight ones with sweet smells, with cheese-bread or sleep, with music or dancing. Fevers occurring every fourth day are cured easily by suddenly startling the unprepared patients, and by means of herbs producing effects opposite to the humours of this fever." It is all very simple and pleasant, but you hardly feel as if it would be a safe place in which to indulge in any very serious illness.

